# Clinical, pathological, and molecular data on desmoplastic/nodular medulloblastoma: case studies and a review of the literature 

**DOI:** 10.5414/NP300205

**Published:** 2016-02-09

**Authors:** Aurore Siegfried, Anne Isabelle Bertozzi, Franck Bourdeaut, Annick Sevely, Najat Loukh, Camille Grison, Catherine Miquel, Delphine Lafon, Nicolas Sevenet, Torsten Pietsch, Christelle Dufour, Marie-Bernadette Delisle

**Affiliations:** 1 Neuropathology, Rangueil, Toulouse University Hospital,; 2 Pediatric Hematology-Oncology, Purpan, Toulouse University Hospital; 3 INSERM U830, Pediatric Oncology, Curie Institute, SiRIC Curie Institute, Translational Research in Pediatric Oncology, Paris,; 4 Neuroradiology, Purpan, Toulouse University Hospital,; 5 Somatic Oncology Laboratory, Curie Institute, Paris,; 6 Neuropathology, Sainte Anne University Hospital, Paris V University, Paris,; 7 Molecular genetics, INSERM U916, Institut Bergonié, Faculty of Pharmacy, Bordeaux, France,; 8 Neuropathology, University of Bonn, Bonn, Germany,; 9 Pediatric and Adolescent Oncology, Gustave Roussy Institute, Villejuif, and; 10 INSERM UMR 1037 and Toulouse III University, Toulouse, France

**Keywords:** desmoplastic medulloblastoma, treatment, prognosis, pathology, genomic

## Abstract

The aim of this study was to better define the clinical and biopathological features of patients with desmoplastic/nodular medulloblastoma (DNMB) and to further characterize this subgroup. 17 children aged < 5 years, with initial DNMB treated according to the HIT-SKK protocol, were evaluated. A retrospective central radiological review, a pathological and immunohistochemical study, and array-CGH and sequencing of germline *SUFU* and *PTCH1* genes were performed. 15 histologically reviewed cases were confirmed as DNMB including three cases of medulloblastoma with extensive nodularity. Median age at diagnosis was 26 months. Radiology showed five cases with a vermis location and one with T2 hyperintensity. All cases showed a SHH immuno-profile. A 9q deletion was found in 6 cases, a *MYCN–MYCL* amplification in 1 case, and a *SUFU* germline mutation in 1 case (/9). The presence of *SUFU* and *PTCH1* germline mutations agreed with previous reports. At 3 years, progression-free survival and overall-survival rates were 72 ± 15% and 85 ± 10%, respectively. The rate of recurrence was relatively high (4 patients). This may have been because chemotherapy was delayed in two cases. Age > 3 years, and residual tumor may also have been an explanation for recurrence.

## Introduction 

Medulloblastoma (MB) is the most common childhood malignant central nervous system tumor. Clinical, pathological, and molecular variables are presently considered to stratify disease risk. The World Health Organization (WHO) classification of central nervous system tumors describes classic MB, desmoplastic/nodular MB (DNMB), MB with extensive nodularity (MBEN), anaplastic MB, and large-cell MB [1]. DNMB, including MBEN, represents 10% of all cases in children, reaching 57% in children aged < 3 years [2]. DNMB shows reticulin free nodules with a distinct neuronal immunophenotype, which are particularly intense in MBEN. Reticulin-rich zones have a high MIB1 index. 

An international meta-analysis has shown that the histology of DNMB is a strong and independent favorable prognostic factor in young children, even in cases with metastatic disease [3]. Prolonged remission can be obtained when these patients are treated with intensive postoperative chemotherapy without irradiation [4]. Globally, progression-free survival (PFS) is higher in this histopathological sub-group [5]. An accurate diagnosis of DNMB is therefore crucial in young children, given the critical consequences related to treatment decisions. 

Recent molecular expression and methylation profiles have led to a classification of MB according to molecular subgroups [6], which have been reduced to four by a consensus: i.e., WNT (the Wingless activated pathway), SHH (the Sonic Hedgehog activated pathway), group 3, and group 4 [7, 8, 9, 10]. The SHH subgroup represents ~ 25 – 30% of all cases of MB, but a much higher proportion of cases in early childhood DNMB (92% in those aged < 2 years) [11, 12]. There is a consistent predisposition to *PTCH1* or *SUFU* germline mutations in MB patients aged < 3 years, with MBEN strongly related to Gorlin’s syndrome [11, 13]. In agreement with the chromosomal location of the *PTCH1* and *SUFU* genes, the loss of 9q and 10q are the most frequent chromosomal alterations found in this group [12, 13]. The SHH pathway may also be activated through activating mutations of *SMO* or amplification at the *GLI2* or *SHH* loci [14]. Comparative array genomic hybridization (array-CGH) can reveal *MYC–MYCN* amplification and most related chromosomal abnormalities [15]. 

Immunohistochemistry may help detect the implicated signaling pathways, including SHH [7]. GAB1, Filamin A, YAP1, and p75NTR are useful markers for the SHH group [16, 17, 18]. Ellison et al. [18] have defined a diagnostic immunohistochemical method to distinguish SHH, WNT, and non-SHH/WNT tumors from formalin-fixed paraffin-embedded tissues. 

In this study, we review our experience with patients aged younger than 5 years with DNMB and treated according to the HIT-SKK 92 trial [4]. Our aim was to assess the clinical, pathological, and biological data from this particular group with MB and to determine the relationships of these data with outcomes in comparison with literature. 

## Materials and methods 

### Patients’ characteristics and treatments

17 children aged < 5 years and with newly diagnosed DNMB/MBEN after surgical excision were evaluated. Staging included pre- and postoperative cranial magnetic-resonance imaging (MRI) or computed tomography (CT), a spinal MRI, and CSF cytology. Available CT and/or MRI scans were centrally reviewed (14 patients). All children were treated according to the HIT-SKK 92 trial, which combined systemic chemotherapy with intraventricular chemotherapy (methotrexate) [4]. 

### Pathological and molecular analyses

An initial diagnosis was made by the local pathologists. The French group of pediatric neuro-oncology pathologists (GENOP) reviewed all cases. Further analyses were done in different laboratories using formalin-fixed paraffin-embedded specimens. Histological preparations were stained with hematoxylin-eosin and reticulin. Immunohistochemistry included synaptophysin, MIB1, INI1/BAF47, and beta-catenin. This was completed using GAB1 (1/100, polyclonal rabbit, Abcam, Cambridge, UK), Filamin A (1/500, clone PM6/317, Chemicon International, Billerica, MA, USA), YAP1 (1/100, clone 63.7, Santa Cruz Biotechnology, Dallas, TX, USA), P53 (1/100, clone DO7, Dako, Glostrup, Denmark), NeuN (1/500, clone A60, Millipore, Billerica, MA, USA), and P75NTR (1/400, clone NGFR5, Thermo, Fremont, CA, USA). 

*MYC* and *MYCN* statuses were collected from fluorescence in-situ hybridization and/or from array-CGH. The array-CGH techniques were performed on two different platforms [15]. Patients with a blood sample and whose parents had given their informed consent for genetic studies were assessed for germline mutations. *SUFU* gene mutational screening used procedures as previously described [11]. *PTCH1*-point mutation analysis was performed using Enhanced Mismatch Mutation Analysis (EMMA), a procedure based on hetero-duplex analysis [19]. 

### Statistical analyses

PFS and overall survival (OS) were calculated using the Kaplan-Meier method. Differences between the groups were assessed using the log rank test. PFS was defined as the time from the date of diagnosis until the date of first progression, death from any cause, or last contact. OS was defined as the time from the date of diagnosis until death from any cause or last contact. 

## Results 

### Patients’ characteristics and treatments (Table 1)

Of the 17 potentially eligible patients, 2 were excluded (case 10 tumor data unavailable, case 13 reclassified as a classic/biphasic medulloblastoma). Median age at diagnosis was 26 months (range 6 – 59), 4 patients were older than 3 years. The male/female ratio was 2 : 1. 

Tumor location was midline in five cases, restricted to one cerebellar hemisphere in six cases, and affected both the midline and hemisphere in three. Five cases showed invasion of the dura mater. In six patients, tumor extension followed the cerebellum folia (gyriform pattern).The tumors were hypo- or isointense in T1 and usually isointense in T2. One patient had metastasis. Complete surgical resection was obtained initially or after a second surgery. In case 9, the second surgery was performed after onset of chemotherapy. Adjuvant chemotherapy began within a median delay of 23 days (range 14 – 119) after the last surgery. 13 patients received three cycles of chemotherapy, and 2 patients received only two cycles because of neurotoxicity (case 17) or an early metastatic relapse (case 5). Two patients did not receive intraventricular methotrexate (cases 5 and 14). 

### Outcomes

**Figure 1.**
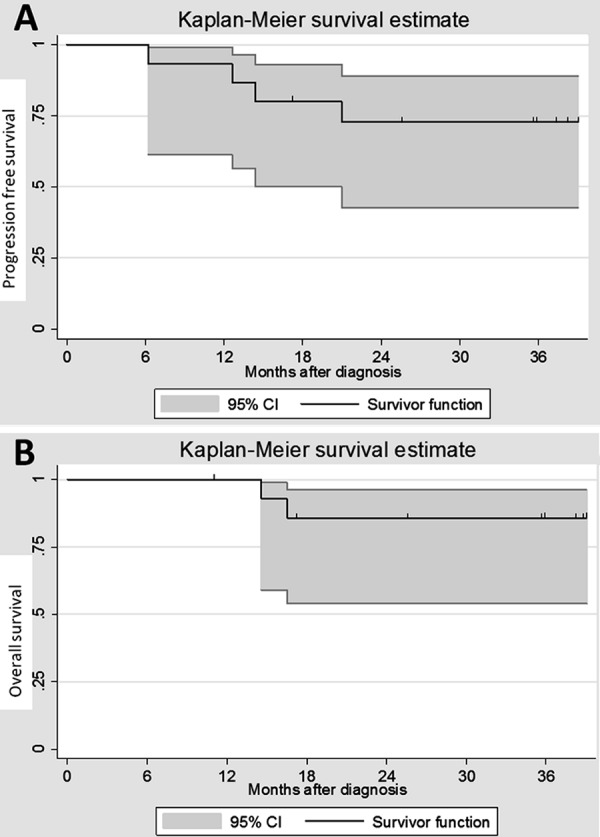
A: Progression free survival. The confidence interval (CI) was defined at 95%. B: Overall survival. The CI was defined at 95%.

The median follow-up was 37 months (range 15 – 50). At 1 year, PFS and OS were 93 ± 8% and 100%, respectively. At 3 years, PFS and OS were 72 ± 15% and 85 ± 10%, respectively (Figure 1). Four relapses occurred at 22 and at 360 days after the end of treatment. Two patients with local (case 9) or a metastatic relapse (case 14) died at 12 months and 15 months after diagnosis, when at 30 and 21 months of age, respectively. Case 5, who had a metastatic relapse, received sequential high-dose chemotherapy and craniospinal irradiation. Case 6, with bi-focal recurrence, was enrolled in a phase I study on SHH inhibitors. Both patients were in complete remission at the last follow-up. 

### Pathologic findings (Table 2)

All 15 retained cases fulfilled the criteria for desmoplastic/nodular features: 3 typical MBENs and 12 DNMBs. Six of these 12 DNMB had large nodules reminiscent of MBEN, but these were rare and showed faint NeuN and synaptophysin expression. The combination of positive GAB1, YAP1, and Filamin A staining indicated an SHH profile in all tumors [18]. All tumors were positive for anti-P75NTR antibody. In case 5, immune staining with GAB1 and YAP1 was faint and focal. The inter-nodular MIB1 index was between 40%, and 80% in 12 cases, and did not exceed 20% in 3 cases (cases 4, 6, 8). P53 expression was low (≤ 10% in all cases). One tumor (case 2) showed positive nuclei for beta-catenin (10%), without a *CTNNB1* mutation after direct sequencing, and chromosome 6 monosomy. 

### Genetic features (Table 2)

**Figure 2.**
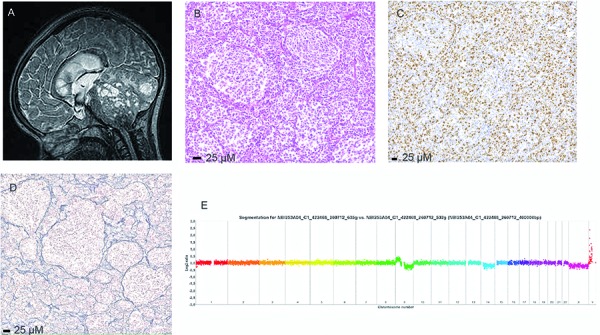
Case 11. DNMB, SHH+. A) T2 MRI: an isointense median mass extending to the 4^th^ ventricle and to the right cerebellar hemisphere. B) HE: clear nodules separated by densely cellular zones. C) MIB1: high index in the internodular zones. D) Reticulin delineated nodules. E) Array-CGH: gain of 9p and loss of 9q.

From the tumor material, array-CGH, *PTCH1*, and *SUFU* direct sequencing were performed for 13, one, and three cases, respectively. Germline *PTCH1* and *SUFU* sequencing was done for seven and nine cases, respectively. From array-CGH, we found evidence of a 9q deletion encompassing the *PTCH1* locus in six cases. An inactivating mutation of *PTCH1* on exon 21 was seen in the tumor DNA, which definitively proved the SHH subtype in one tumor (Figure 2, case 11). In two cases where 9q was deleted (6, 16), *PTCH1* germline mutations on exons 6 and 17, respectively, were found associated with a neutral variant, but were considered as polymorphisms. In another deletion case (1), an isolated silent mutation was found. *PTCH1* germline alterations were found in case 7, without a 9q deletion, but both variants were considered to be polymorphisms. We did not observe any 10q deletions, whereas a germline *SUFU* mutation was observed in only one of the nine analyzed cases. No *GLI2* or *SHH* amplifications were observed. One tumor (case 5) showed *MYCN* and *MYCL* amplifications, and a 9q deletion encompassing *PTCH1;* no chromothripsis was seen in that tumor. No tumors had chromosome 17q gain or isochromosome 17q, these being the most frequent chromosomal alterations in infant non-desmoplastic, non-SHH MB. 

## Discussion 

This study reports on a relatively small number of cases but highlights several points. Because of the relatively low number of patients, our findings are discussed without making a definitive conclusion. Our findings led us to review the literature on the desmoplastic/nodular sub-group of MB, which was almost always linked to the SHH pathway at a young age. 

DNMB was identified as an independent favorable prognostic factor in infants [3, 5, 20]. In the HIT-SKK 92 trial, the 3-year PFS and OS were 85 ± 8% and 95 ± 5%, respectively [4]. In the present study, at 3 years, the PFS and the OS were 72 ± 15% and 85 ± 10%, respectively, which was not as good as in prior published studies, with recurrence occurring in 4 patients. The delay in starting chemotherapy exceeded 4 weeks in 2 of these 4 recurring patients. T2 hyperintensity was noted in 1 of these 2 patients. Age at diagnosis was > 3 years in 2 patients who relapsed; there were 4 patients aged > 3 years in the series. In another relapsing patient, who died, there was a tumoral residue after initial surgery. Delay starting chemotherapy, age at diagnosis, and/or a residual tumor should be considered as potential outcome factors. 

Location of the lesions was not restricted to the cerebellar hemisphere: 5/14 children presented with a midline tumor. Liu et al. [21] found that all patients with DNMB (12/12 cases) presented with a tumor involving the vermis. Although cerebellar hemispheric tumors are predominant in the SHH subtype [22], a recent study has shown that SHH subtypes were not exclusively hemispheric and that hemispheric MBs were not always SHH-activated [23]. In another study, nearly 80% of SHH tumors were located in the vermis when the group was restricted to infants [24]. Location of the tumor varies depending on age, pathology, and molecular subtype. A midline location is often seen, especially in younger patients with a combined desmoplastic/nodular and SHH classification. 

DNMB can express the nodular phenotype to varying degrees. Desmoplasia may occur in any MB variant as a normal reactive phenomenon when tumor cells invade the leptomeninges. However, so far, sub-classification has been difficult [20]. Indeed, one MB was reclassified as a classic MB with nodules (biphasic MB). The reticulin stain, delineating pale islands, remains a strong diagnostic feature [20] and immunohistochemistry may be helpful. The association between DNMB and activation of the SHH signaling pathway is not constant but is particularly elevated in infants [12]. Here, SHH immunohistochemistry was absent in excluded biphasic MB arguing again for the usefulness of this procedure. 

The P53 index was low. *TP53* gene status was not studied. A worse prognosis has been associated with a *TP53* mutation in SHH MB patients aged > 5 years [25]: in contrast, all patients were aged < 5 years in our series. 

*MYC* gene amplification was absent within our cohort. The only *MYCN* amplification was found in a case with a 9q deletion encompassing the *PTCH1* locus, which strongly suggests this tumor belongs to the SHH subgroup. The association of *MYCN* and *MYCL* in this tumor is a striking and unique observation, although isolated *MYCL* amplifications have been sporadically observed in SHH tumors [26] and *MYCN* is one of the most frequently amplified genes in SHH tumors [9]. Multiple amplicons in a SHH setting usually associate *GLI2* with *MYCN* and may indicate Li-Fraumeni syndrome, which is linked to a dismal prognosis. If though the status of *MYCN* amplification in MB as an outcome indicator remains globally controversial, it has been suggested that *MYCN*-amplified SHH tumors have a poorer outcome [2, 22]. Retrospectively, one may wonder if tumors with multiple amplicons and/or *MYCN*-SHH tumors justify a more straightforward and intensive treatment. 

Cases of MB are known to occur in predisposition syndromes, particularly in Gorlin’s syndrome [13, 27, 28]. Germline *SUFU* mutations have been found to be responsible for a high proportion of desmoplastic MB in children aged < 3 years [11]. These mutations may cause Gorlin’s syndrome with a higher risk of developing MB than *PTCH1* mutations [28]. It has been shown that MBEN is strongly associated with Gorlin’s syndrome, particularly in patients aged < 3 years [13, 27]. In our series, the frequency of *SUFU* mutations (1/6 tested patients aged < 3 years) was in concordance with that reported in the literature. The absence of a deleterious germline *PTCH1* mutation reinforces the hypothesis that desmoplastic MB as a stand-alone clinical sign is a low-evidence criterion for screening for a deleterious germline *PTCH1* mutation [28]. 

This study confirms the prognostic clues for MB subtypes, especially desmoplastic types, depending on the complementary data. The efficiency of a targeted therapy, which can be particularly useful in these tumors, relies on combining criteria from pathology and genomics [10]. Further large international studies are needed to better comprehend this childhood disease. 

## Acknowledgments 

The authors thank E. De Carli, C. Icher, C. Chappé, C. Berger, and S. Thouvenin-Doulet for the clinical data, P. Varlet, E. Uro-Coste, A. Rousseau, M. Peoc’h, S. Eimer, and D.C. Chifforeanu for the tumor material, A. Jouvet, D. Figarella-Branger, and C. Godfraind (GENOP), the geneticists from the Curie and G. Roussy institutes, and from Bergonié Institute, H. Collineau for statistical analyses, and Clinical Research Assistants (Paris, Toulouse, France). 

## Conflict of interest statement 

The authors declare no conflict of interest for this study. 

**Table 1.** Demographic, radiological and treatment data.

Clinical case number	Age (years)/gender	Location of tumor	DM/M status	MRI T1	MRI T2	Extent of first surgery	Delay before chemotherapy (days)	Last follow up
1	4.9/M	LR	P/M0	Hypo	Iso	R0	14	CR1
2	2/F	LL	A/M0	Hypo	Iso	R1	30	CR1
3	1.2 /M	MED	A/M0	Iso	Iso	R1	107	CR1
4	2.2/M	MED	A/M0	Iso	Iso	R0	15	CR1
5	4/M	LL	P/M0	Hypo	Hyper	R0	90	CR2
6	3/F	MED, LL	A/M0	ND	Iso	R1	119	CR2
7	1.9/F	MED	A/M0	ND	Iso	R0	22	CR1
8	3.2/F	LL	P/M+	Iso	Iso	R0	30	CR1
9	1.5/M	MED, LR	P/M0	Iso	Iso	R1	13	D
**10**	**3.5/F**	**ND**	**ND**	**ND**	**ND**	**R0**	**30**	**CR1**
11	4.3/M	LR	A/M0	Iso	Iso	R0	20	CR1
12	1/F	MED, LR	P/M0	Iso	Iso	R1	2	CR1
**13**	**3.5/M**	**MED**	**A/M0**	**Hypo**	**Hyper**	**R1**	**17**	**CR2**
14	0.5/M	MED	A/M0	Iso	Iso	R0	19	D
15	2.1/M	ND	ND	ND	ND	R0	30	CR1
16	0.8/M	MED	A/M0	Iso	Iso	R1	35	CR1
17	2.5/M	LR	A/M0	Iso	Iso	R0	20	CR1

M = male; F = female; LR = lateral right; LL = lateral left; MED = medial; DM = dura mater; P = presence of DM invasion; A = absence of DM invasion; M0 = no metastasis detected at diagnosis; M+ = metastasis detected at diagnosis; Hyper = hyperintensity; Hypo = hypointensity; Iso = isointensity; R0 = no residue; R1 = residual tumor; CR1 = complete remission after HIT-SKK protocol; CR2 = secondary complete remission; D = death; ND = not done. **Bold** = excluded cases.

**Table 2.** Histopathology, immunochemistry and molecular profiling.

Clinical case number	Morphological subtype	Immunohistochemistry					Chromosome 9q copy number changes on a-CGH	PTCH1 sequencing	SUFU sequencing
		Gab1	P75 NTR	Fil A	YAP1	P53			
1	DNMB	+	+	+	+	< 3%	Chr 9 del	Wild type (germline)	Wild type (germline)
2	MBEN	+	+	+	+	< 5 %	–	ND	Wild type (germline)
3	DNMB	+	+	+	+	10%	–	ND	Mutation (germline)
4	DNMB	+	+	+	+	10%	–	Wild type (germline)	Wild type (germline)
5	DNMB	+	+/–	+/–	+	0%	9q del	Wild type (germline)	Wild type (germline)
6	DNMB	+	+	+	+	5 – 10%	9q del	Variant (germline)	Wild type (germline)
7	DNMB	+	+	+	+	5 – 8%	–	Variant (germline)	Wild type (germline)
8	MBEN	+	+	+	+	5%	ND	ND	ND
9	DNMB	+	+	+	+	0%	9q del	ND	Wild type (germline)
**10**	**DNMB**							**Wild type (germline)**	
11	DNMB	+	+	+	+	5%	9q del	Inactivating Mutation (tumoral)	Wild type (germline)
12	DNMB	+	+	+	+	10%	–	ND	ND
**13**	**CMB**	**+/–**	**–**	**+**	**+/–**	**5%**	**–**	**Wild type (germline)**	**Wild type (germline)**
14	MBEN	+	+	+	+	0%	ND	Wild type (germline)	ND
15	DNMB	+	+	+	+	< 5%	–	ND	Wild type (tumoral)
16	DNMB	+	+	+	+	< 5%	9q del	Variant (germline)	Wild type (tumoral)
17	DNMB	+	+	+	+	< 5%	–	ND	Wild type (tumoral)

DNMB = desmoplastic-nodular medulloblastoma; MBEN = medulloblastoma with extensive nodularity; CMB = classic medulloblastoma; Fil A = Filamin A; a-CGH = array Comparative Genomic Hybridization; ND = not done. Immunohistochemistry: + = homogeneous stain in internodular zone; +/– = focal and heterogeneous stain. Molecular biology: del = deletion; germline: DNA extracted from lymphocytes; tumoral: DNA extracted from tumor specimen. **Bold** = excluded cases.
